# Childhood adversity and acute stress vulnerability at adulthood: The mediating role of sleep

**DOI:** 10.1192/j.eurpsy.2023.2348

**Published:** 2023-07-19

**Authors:** R. Admon

**Affiliations:** Psychology, University of Haifa, Haifa, Israel

## Abstract

**Introduction:**

Numerous preclinical and clinical studies established the contribution of childhood adversity to acute stress vulnerability at adulthood. Several different physiological, psychological and behavioral factors have been suggested as putative mediators of this association. Sleep, and more specifically sleep disruption, has emerged as one such promising candidate. First, adverse childhood experiences have been repeatedly associated with adult sleep disorders. Second, individuals that suffer from stress-related psychopathology at adulthood often exhibit sleep disturbances, to the extent that sleep difficulties are diagnostic criteria for many of these disorders. Third, inefficient sleep pre and post exposure to acute stress was shown to increase the likelihood for maladaptive outcome, potently by impairing critical processes that occur during sleep as arousal regulation and memory consolidation.

**Objectives:**

To date, very few studies integrated these independent lines of research. Also, previous studies mostly assessed sleep via self-reported diary or a single night measurement at laboratory settings. The current study aimed to provide a more ecological and continuous account for sleep patterns, and their putative associations with childhood adversity on the one hand and vulnerability to acute stress at adulthood on the other hand.

**Methods:**

Ninety-six healthy adult female participants completed the well-established childhood trauma questionnaire (CTQ) before wearing a wearable sensor for seven consecutive days and nights while maintaining their regular life routine. Following that, participants all underwent an acute laboratory stress induction procedure while their psychological and endocrine responses were recorded at multiple time points throughout.

**Results:**

Sleep patterns fully mediated the association between childhood adversity and psychological response to acute stress at adulthood (Figure 1). Specifically, elevated levels of childhood trauma were associated with more variation in sleep duration across the recording period, which in turn was associated with higher stress-induced negative affect. Interestingly, this association did not emerge with respect to mean sleep time nor with stress-induced cortisol release.

**Conclusions:**

Results imply that childhood trauma may lead to irregular sleep patterns which in turn contribute to exaggerated emotional response to acute stress at adulthood. These findings support the mediating role of sleep in the link between childhood adversity and acute stress vulnerability at adulthood, and highlight sleep as a viable target for early or even preventive intervention.

**Disclosure of Interest:**

None Declared

